# Benign Metastasizing Leiomyoma of the Uterus with Pulmonary and Bone Metastases

**DOI:** 10.1155/2021/5536675

**Published:** 2021-06-08

**Authors:** Parikshit Padhi, Margarita Topalovski

**Affiliations:** ^1^Memorial Medical Center, Department of Hematology Oncology, 2530 S. Telshor Blvd, Las Cruces, NM 88011, USA; ^2^Memorial Medical Center, Department of Pathology, 2540 S. Telshor Blvd, Las Cruces, NM 88011, USA

## Abstract

Benign metastasizing leiomyoma (BML) is a rare spindle cell neoplasm seen in middle-aged women who have a history of leiomyoma of the uterus. The most common sites of metastases are the lungs; however, other sites of spread have been documented. These tumors by definition have no malignant features on histology and tend to be estrogen and progesterone positive. We present a middle-aged woman who was incidentally found to have multiple pulmonary nodules and a mass on her sternum after she was involved in a motor vehicle accident. She had a history of uterine leiomyoma and had undergone a hysterectomy ten years prior to the accident. Biopsies were performed of the lung nodules and sternum mass and compared to her hysterectomy specimen, and they were identical, and hence, she was diagnosed with BML. Due to the growing tumor of her sternum, she was started on tamoxifen with stability of her tumors. These tumors, since they are benign, tend to have an indolent course. However, in the instances that treatment is warranted, options include surgery or antiestrogen therapy. We will be discussing the pathogenesis, histological findings, and treatment options of this rare condition. Our case is unique because BML in general do not tend to spread to multiple organs and tend to be limited to one site of disease.

## 1. Introduction

Benign metastasizing leiomyoma is a rare, benign, spindle cell neoplasm usually diagnosed in middle-aged women who have a history of uterine leiomyoma. The most common site of metastases is the lung although other sites such as the heart, bones, and lymph nodes have been reported. Although these tumors are metastatic deposits from the uterus, they have no malignant features morphologically or clinically and most have an indolent course. We present a young female with BML to the lungs and bones, and we will discuss about BML, its pathogenesis, and treatment options.

## 2. Case Presentation

**Figure 1:**
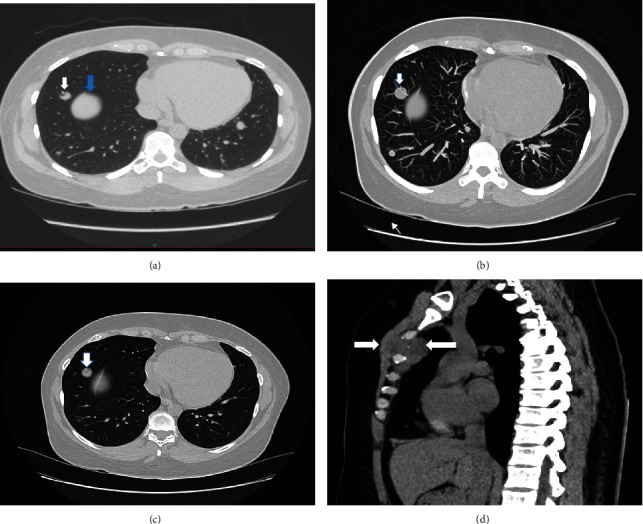
Initial CT scan at the time of motor vehicle accident in November 2019 (a) shows multiple pulmonary nodules the largest measuring 1.2 cm (white arrow) in the anterior right lower lobe (RLL). The blue arrow in the CT scan shows the top of the liver. In March 2020, CT scan of the chest (b) showed the dominant RLL lesion now measuring 2.0 cm depicted with the thicker arrow and a new smaller posterior RLL nodule depicted with the thinner arrow. After starting tamoxifen, the CT scan of the chest in November 2020 (c) shows stability of the dominant anterior RLL measuring 1.8 cm and the posterior RLL nodule is improving. (d) Is the sagittal image of the chest CT in March 2020 revealing the sternum mass that measured 5.7 × 3 cm with invasion into the cortex of the bone causing destruction of the bone.

We present a 47-year-old woman with no medical history, who presented to the hospital after a motor vehicle accident. She has a history of leiomyoma of the uterus and underwent a total abdominal hysterectomy at age 37. While undergoing routine X-rays and Computed Tomography (CT) scans of her chest, abdomen, and pelvis, she was found to have multiple pulmonary nodules in both lungs, largest in the right lower lobe measuring 1.2 cm (Figure 1(a)). She is a nonsmoker and has never had any prior lung infections. As these nodules were thought to be benign, it was decided to repeat imaging in 3 months. Two months after her accident, she complained of swelling around her manubrium. An ultrasound of the sternal area found a 1.2 cm mass arising from the manubrium sternum. Due to COVID-19, there were delays in her care. Four months after initial presentation, she underwent a Positron Emission Tomography (PET scan) that redemonstrated the multiple pulmonary nodules with the largest nodule now measuring 1.4 cm with a standardized uptake value (SUV) of 1.4. She first presented to our cancer center two months after the PET scan. We repeated a chest CT which showed new bilateral lung nodules, and the largest right lower lobe nodule now measured 2 cm (Figure 1(b)). The mass in her manubrium increased to 5.7 × 3.0 cm and was invading into the cortex of the bone (Figure 1(d)). A biopsy of the right lower lobe lung mass and manubrium soft tissue mass was performed.

**Figure 2:**
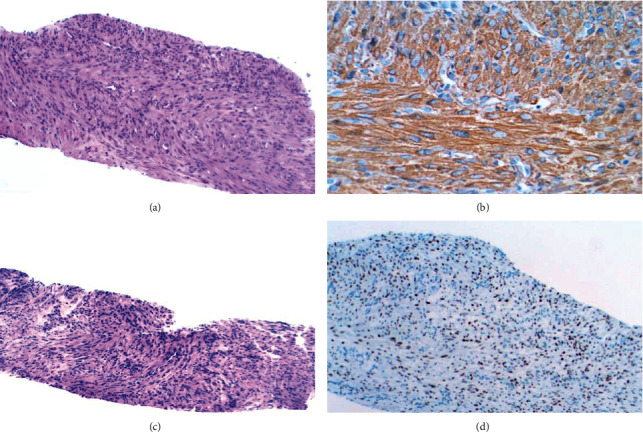
Lung biopsy H&E stain (a) shows a spindle cell neoplasm with no atypia, necrosis, or increased mitoses that is positive for smooth muscle actin (b). The sternum biopsy (c) also histologically resembles the lung biopsy and shows a spindle cell neoplasm that did not have any malignant features. The sternum biopsy is positive for estrogen receptors (d) and progesterone as well. Since no malignant features were seen in either biopsy and given history of leiomyoma, a diagnosis of benign metastasizing leiomyoma was given.

The lung biopsy revealed a smooth muscle neoplasm that was positive for smooth muscle actin, vimentin, calponin, and desmin. Ki67 was 1-2%. It was negative for AE1/AE3 and p63 and showed no atypia, increased mitoses, or necrosis going against a metastatic leiomyosarcoma. This was compared to her hysterectomy specimen and histologically was identical. A diagnosis of benign metastasizing leiomyoma (BML) was made. The sternum biopsy showed a similar spindle muscle neoplasm that was positive for smooth muscle markers. Ki67 was 5% and was histologically similar to the prior lung biopsy and hysterectomy specimen. Estrogen receptor (ER) and progesterone receptor (PR) staining was performed and was strongly positive on both the lung and sternum biopsies. After a second consultation, it was confirmed as BML with no evidence of malignant sarcoma (Figure 2).

Although there was concern for sarcoma, we had no evidence histologically and hence, it was confirmed she had BML involving the bone and lungs. As she was symptomatic from the sternum mass, we decided to pursue with treatment. We reviewed case reports and discussed her case in our multidisciplinary tumor board after which we initiated endocrine therapy. She was initiated on tamoxifen, and after three months, a repeat chest CT showed stability of the size and number of the lung nodules as well as stability of her sternum mass (Figure 1(c)). Given that the lesions stopped growing after tamoxifen, it favored BML rather than a sarcoma. The plan is to continue with tamoxifen for now with plans for oophorectomy or GnRH analog and aromatase inhibitors if her disease worsens in the future.

## 3. Discussion

Benign metastasizing leiomyoma (BML) is a rare entity with the first case being reported by Dr. Steiner in 1939, in which he reported a contradictory case of a young woman who had benign appearing multiple lung nodules suggestive of a benign metastasizing fibroleiomyoma [1]. There have been approximately 130 reported cases since then. BML most commonly occurs in middle-aged women in the perimenopausal period. All patients have a history of uterine leiomyoma (ULM), with most women having had a hysterectomy. Most patients are asymptomatic, and the tumors are incidentally found when imaging studies are performed for unrelated reasons. The most common metastatic site is the lung; however, sites such as the heart, retroperitoneal lymph nodes, skin, and bones have been reported [2–4]. As mentioned above, most cases are asymptomatic; however, patients may complain of chest pain, shortness of breath, or cough. In our case, our patient was initially asymptomatic but later developed a painful soft tissue chest mass. Kayser et al. reported an average time of 14.9 years from time of hysterectomy to development of BML; however, Barnas et al. reported a median time of just under 9 years between hysterectomy and development of BML [5, 6]. Another study reported a median age of 46 years of which 82.6% of the patients had undergone a prior hysterectomy for ULM, with a median time from surgery to onset of BML being 10.5 years [7].

The pathogenesis of BML is not truly known. One theory is that there is hematogenous spread of benign endometrium cells into organs during the time of hysterectomy for ULM. Another theory is that they are leiomyosarcomas with low-grade malignant potential; as more cases are being reported, it seems unlikely that they are primary leiomyomas of the lung given the similarity between the uterine tumor and the metastatic site [8]. Patton et al. reported a possible X-linked clonal inactivation suggesting that there is a monoclonality to this tumor [9]. They also mention that telomere lengths do not play a role in this entity. Chromosomal abnormalities have been detected in BML. Nucci et al. described abnormalities in 19q and 22q while Lee et al. noted abnormalities in both the lung tumors and uterine tumors with translocations (12;14) and (1;2) noted [10, 11]. Abnormalities in chromosomes 1, 7, 13, and 14 have been reported as well [12]. Mutations in BMP8B and MED12 genes have been identified, and mutations in ARID2 and NTRK1 and amplifications in BC11B and TCL1A have been noted too [13].

Microscopic findings of BML demonstrate characteristics of smooth muscle proliferation and tend to stain positive for actin, desmin, or vimentin. They do not to have a high mitotic rate, high Ki67, atypia, high degree of cellularity, or poor differentiation suggesting that they are benign tumors [3]. They tend to be strongly positive for estrogen and progesterone, suggesting that they originate from the gynecological tract [14–16].

There are no standard treatment guidelines for BML. Since most cases are asymptomatic and tend to be slow-growing, they can be observed unless symptoms develop. Surgical resection of metastatic site if feasible would be the preferred treatment modality [17]. Given that these tumors are estrogen and progesterone positive, they tend to respond to endocrine therapy. Spontaneous regression during pregnancy and menopause has been noted suggesting that once patients attain menopause, there could be regression of these tumors [18]. In nonresectable metastatic disease, manipulation of the hormones either via oophorectomy, GnRH analogs, ovarian ablation, tamoxifen, or aromatase inhibitors can be used. With endocrine therapy, most patients have stability of their masses or regression of the tumors. Most cases of BML have been reported in case reports; there has been no long-term follow-up of these patients to know if this entity can be fatal.

## 4. Conclusion

Benign metastasizing leiomyoma is a rare condition usually seen in middle-aged perimenopausal women. Most women have a history of leiomyoma of the uterus with a median time to onset of BML approximately 10 years after definitive hysterectomy. Although these tumors by definition are metastatic deposits, they do not have any malignant features and tend to be slow-growing and asymptomatic. In our case, we presented a perimenopausal woman who developed BML ten years after undergoing a hysterectomy. She had a progressively growing sternum lesion, and biopsy showed BML, and no carcinoma was seen. These tumors tend to be estrogen and progesterone positive suggesting a gynecological origin. Treatments include observation, surgery, or antiestrogen therapy. In our case, we initiated the patient on tamoxifen and this has stopped the growth of her tumors and we plan to continue tamoxifen indefinitely for now.

## Data Availability

All the data is in the main manuscript including the images and details of the cases.
